# Human pancreatic islet miRNA-mRNA networks of altered miRNAs due to glycemic status

**DOI:** 10.1016/j.isci.2022.103995

**Published:** 2022-02-26

**Authors:** Alexandros Karagiannopoulos, Jonathan L.S. Esguerra, Morten G. Pedersen, Anna Wendt, Rashmi B. Prasad, Lena Eliasson

**Affiliations:** 1 Islet Cell Exocytosis, Lund University Diabetes Centre, Department of Clinical Sciences-Malmö, Lund University, CRC 91-11, Box 50332, 202 13 Malmö, Sweden; 2 Clinical Research Centre, Skåne University Hospital, CRC 91-11, Box 50332, 202 13 Malmö, Sweden; 3 Department of Information Engineering, University of Padova, Padua, Italy; 4 Genomics, Diabetes and Endocrinology, Lund University Diabetes Centre Department of Clinical Sciences-Malmö, Lund University, Malmö, Sweden

**Keywords:** Endocrinology, Omics

## Abstract

MicroRNAs (miRNAs) are short non-coding RNAs that regulate gene expression via mRNA targeting, playing important roles in the pancreatic islets. We aimed to identify molecular pathways and genomic regulatory regions associated with altered miRNA expression due to glycemic status, which could contribute to the development of type 2 diabetes (T2D). To this end, miRNAs were identified by a combination of differential miRNA expression and correlation analysis in human islet samples from donors with normal and elevated blood glucose levels. Analysis and clustering of highly correlated, experimentally validated gene targets of these miRNAs revealed two islet-specific clusters, which were associated with key aspects of islet functions and included a high number of T2D-related genes. Finally, *cis*-eQTLs and public GWAS data integration uncovered suggestive genomic signals of association with insulin secretion and T2D. The miRNA-driven network-based approach presented in this study contributes to a better understanding of impaired insulin secretion in T2D pathogenesis.

## Highlights

• Network-based islet miRNA approaches provide a better insight into T2D pathogenesis
• Islets from healthy and hyperglycemic donors have distinct miRNA profiles
• Differentially expressed miRNAs regulate gene networks that affect islet function
• Specific miRNA eQTLs overlap with insulin secretion and T2D genetic markers

## Graphical abstract

**Figure:**
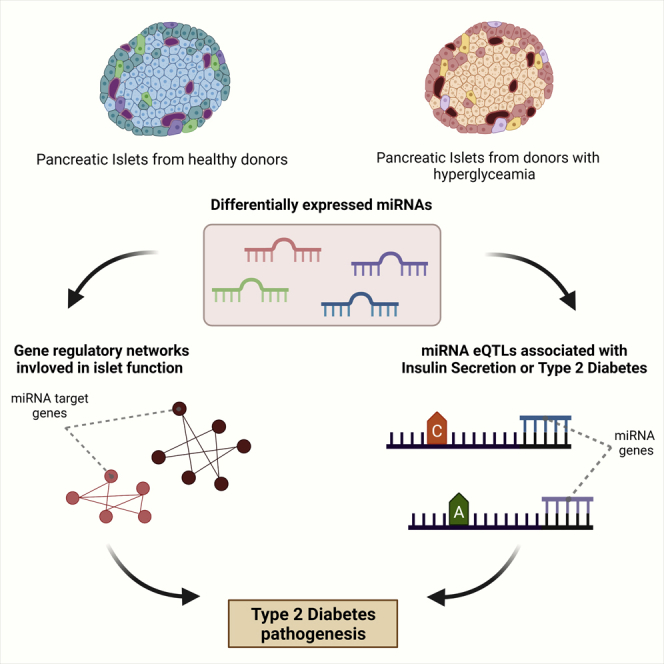

## Introduction

miRNAs are short (≈19–23nt) endogenous non-coding RNAs which most of the time are responsible for silencing genes through inhibition of their translation or destabilization of their target mRNA molecule (Bartel, 2009). However, some cases of upregulation of the miRNA target genes have also been reported (Vasudevan et al., 2007). They have important regulatory roles in various biological processes, from specifying cell identity during development, to fine-tuning cellular functions in response to environmental stimuli. Indeed, it is estimated that 60% of mammalian protein-coding genes are post-transcriptionally regulated by miRNAs (Friedman et al., 2009). Unsurprisingly, perturbed miRNA expression in different tissues has become a recognized feature in human disease pathophysiology (Paul et al., 2018).

Type 2 diabetes (T2D) is characterized by impairment in the regulation and utilization of blood glucose as an energy source in the body. Contributing factors of the pathogenesis is the combination of insulin resistance, in which target tissues lose the capacity of properly responding to insulin, and the dysfunction of pancreatic β-cells, which secrete suboptimal levels of insulin (DeFronzo et al., 2015; Lebovitz, 1999). The functional impairment of glucagon-secreting pancreatic α-cells could also be responsible for the progression of the disease, because glucagon counteracts the effects of insulin and is crucial for maintaining the glucose homeostasis (Wendt and Eliasson, 2020). The importance of miRNAs in the pancreatic islet cells has been unequivocally shown in knockout mouse models of specific miRNAs or via global ablation of miRNA expression by deleting the Dicer1 enzyme that is involved in miRNA maturation (Kalis et al., 2011; Lynn et al., 2007; Martinez-Sanchez et al., 2015; Melkman-Zehavi et al., 2011). Such studies demonstrate that miRNAs can be implicated in events leading to T2D onset. In T2D, β-cell compensation is important, with some miRNAs exerting compensatory effects and others impacting insulin secretion through miRNA-mediated dysfunction (Eliasson and Regazzi, 2020). Among specific islet miRNAs shown to be implicated in dysfunctional β-cell function are miR-7, miR130a/130b, miR-152, miR-184, and the miR-200 family (Eliasson and Regazzi, 2020). In islets, most studies so far have investigated the role of single miRNAs and have not used a network approach of differentially expressed miRNAs and their targets. However, we and others suggest that islet function is regulated by miRNA groups rather than single miRNAs (Eliasson and Esguerra, 2020; Wong et al., 2021).

Genome-wide association studies (GWAS) have been effective in connecting genomic regions to various phenotypes, including T2D and related glycemic traits (Mahajan et al., 2014; Morris et al., 2012). The fact that individual T2D-associated variants have a small contribution to the overall disease risk (Fuchsberger et al., 2016) and that the vast majority of them are located in non-coding regions (Mahajan et al., 2018), makes the association between the variants with specific proteins and pathways complicated. Nevertheless, the identification of disease-specific GWAS variants that are related to the expression levels of proximal genes, or expression quantitative trait loci (eQTL), enables formulation of hypotheses about specific proteins/pathways (Westra and Franke, 2014).

In this study, we aimed to discover human islet miRNAs that are affected due to changes in the glycemic status and investigate their targeting properties by retrieving a list of validated gene targets that are expressed in human islets. Moreover, we identified pathways of the differentially expressed miRNAs and their targets, their functional annotation, and their correlation with different phenotypic traits. Finally, we investigated genomic regions that are associated with miRNA expression via *cis*-eQTL analysis and found SNPs linked to T2D and insulin secretion traits.

## Results

### Global miRNA profiling of human pancreatic islets

Human islets from 18 donors (Table S1) were profiled for 840 miRNAs using Exiqon’s LNA (locked nucleic acid)-based array platform. In total, 470 miRNAs were expressed in our islet samples (Table S2).

**Figure 1:**
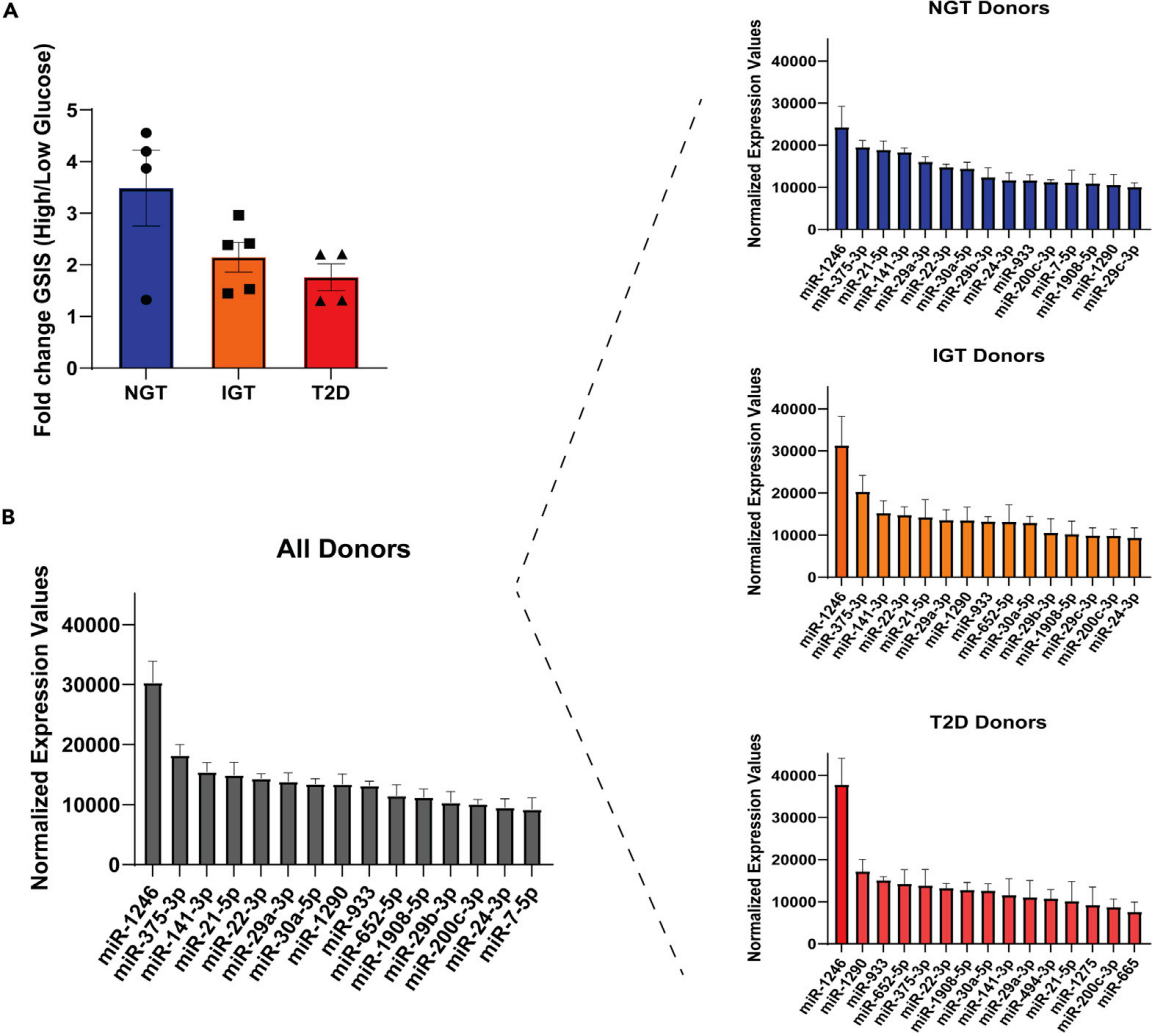
Global miRNA profiling of human pancreatic islets in islets from donors with normal glucose tolerance (NGT), impaired glucose tolerance (IGT), and diagnosed with T2D (T2D) (A) Fold change (high/low concentration glucose) glucose-stimulated insulin secretion (GSIS); low glucose = 1 or 2.8 mmol/L, high glucose = 16.7 or 20 mmol/L. GSIS data are available for a subset of islets included in this study and represent data derived from a larger islet cohort originally presented in (Rosengren et al., 2012). (B) miRNA expression level of the 15 most abundant miRNAs among all, NGT, IGT, and T2D donors. Data are presented as mean ± SEM.

The donors were divided into three groups according to their HbA1c levels. The HbA1c values of the 18 donors ranges from 4.6% to 7%, seven had normal glucose tolerance (NGT) (HbA1c<6%), six had impaired glucose tolerance (IGT) (HBA1c range: 6.0%–6.4%), while five of them were also diagnosed with T2D (HbA1c range: 6.2%–7.0%) (Table S1). Glucose-stimulated insulin secretion (GSIS) data were available for four NGT, five IGT, and four T2D of our islet preparations. Data indicate a decreased trend regarding the function of the IGT and T2D islets compared to the NGT islets (Figure 1A).

By analyzing probes that were captured by all donors and excluding those that corresponded to more than one human miRNAs, the remaining 269 miRNAs showed a large variation regarding their expression levels with only a few miRNAs being highly expressed (Figure S1). Among the 15 most highly expressed miRNAs, we observed both miRNAs that were previously described as highly abundant in human islets or β-cells such as miR-375, miR-141-3p, and members of miR-29, miR-200, and miR-7 families (Kameswaran et al., 2014; Locke et al., 2014) and miRNAs not previously shown to be highly abundant, such as miR-1246, miR-1290, and miR-1908 (Figure 1B). Despite the slight differences in their expression levels and ranking, 11 out of the 15 most highly expressed miRNAs across all donors were still abundant in islets derived from NGT, IGT, and T2D donors (Figure 1B).

Using transcriptome data from the same donors derived from a previous study (Asplund et al., 2020), we sought to reveal correlations between miRNA expression and gene expression of the major islet hormones, namely insulin, glucagon, and somatostatin. The analyses showed 19 miRNAs to be significantly correlated with insulin, 7 with glucagon, and 24 with somatostatin expression (p<0.05), while miR-27b-5p was correlated with the expression of both insulin and glucagon and miRNAs miR-21-3p and miR-497-5p to both glucagon and somatostatin (Table S3).

### Altered expression of miRNAs due to glycemic status

**Figure 2:**
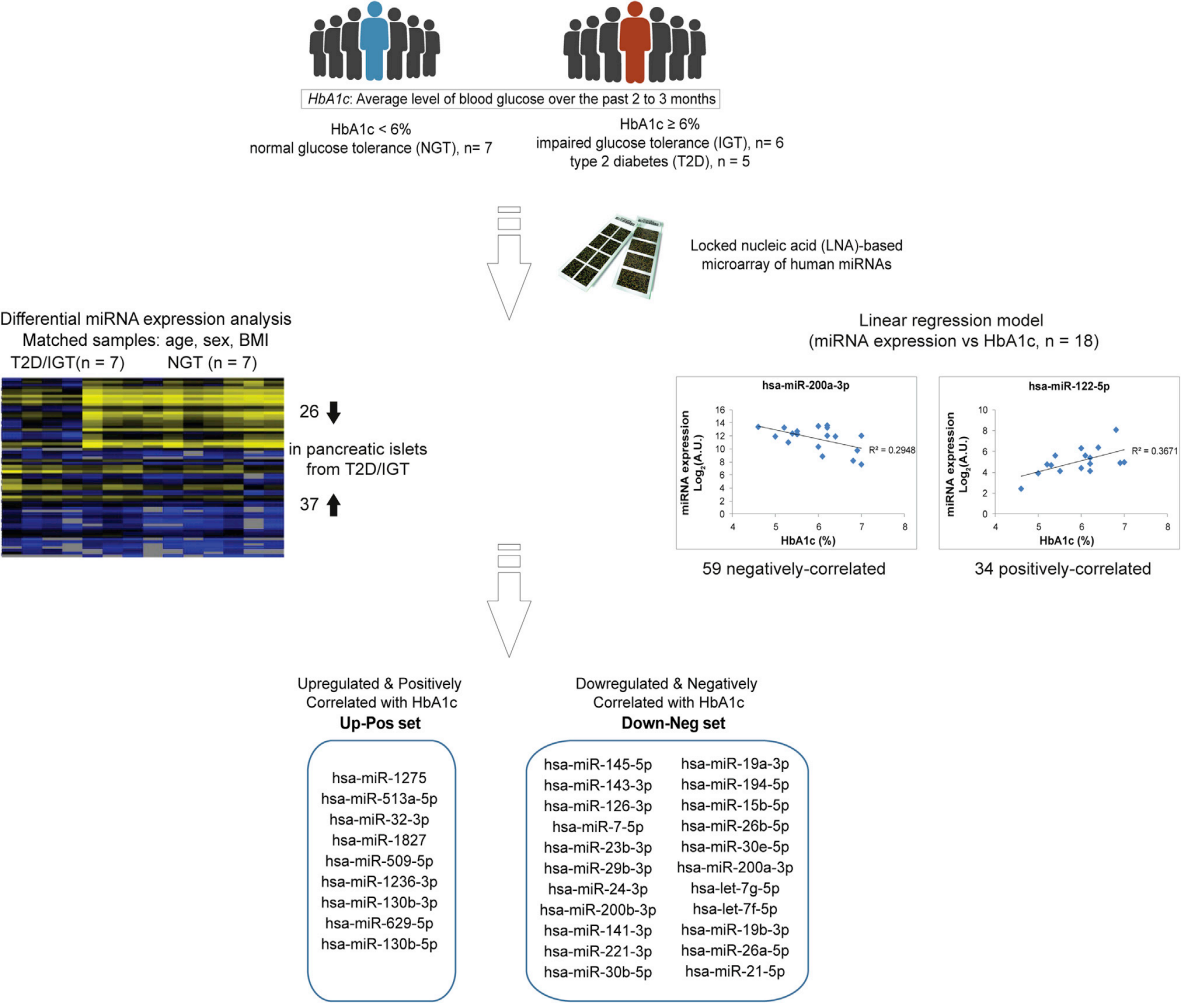
Identification of miRNAs with altered expression due to glycemic status After donors were matched according to age, sex, and BMI, differentially regulated miRNAs were recorded using Significance Analysis of Microarrays (SAM). In parallel, correlations of miRNA expression values with HbA1c levels were performed. Intersection of the significantly regulated and correlated miRNAs revealed a set of upregulated and positively correlated miRNAs (*Up-Pos*) with HbA1c and a set of downregulated and negatively correlated miRNAs with HbA1c (*Down-Neg*).

The workflow for the identification of islet miRNAs with differential expression due to glycemic status of the donors can be seen in Figure 2. First, we matched the NGT donors with the IGT/T2D donors for age, gender, and BMI. The classification enabled the differential expression analysis of miRNAs between the two paired groups using Significance Analysis of Microarrays (SAM) based on false discovery rate (FDR) statistics within the TM4 platform (Saeed et al., 2003) (Figure 2, Table S4). The analysis revealed 37 upregulated and 26 downregulated miRNAs in islets from the IGT/T2D donors compared with NGT donors (FDR<0.1). Among the upregulated miRNAs were miR-1275, miR-32-3p, and miR-130b-5p/3p and among the downregulated we found miR-126-3p, miR-7-5p, and miR-200a.

Next, individual miRNA levels in all 18 donors were associated with HbA1c levels to assess how the *in vivo* long-term glucose exposure affects islet miRNA regulation. For this, we used a linear regression model and adjusted for age, gender, BMI, and diabetic status of the donors, as well as days of culture *in vitro* of the islets. This resulted in 59 miRNAs negatively correlated and 34 miRNAs positively correlated (FDR<0.1) with HbA1c (Table S4). Specifically, we could detect miR-23b, miR-7, and miR-484 to be correlated with HbA1c. All three are miRNAs previously shown to be regulated by glucose (Tang et al., 2009).

Finally, as described in Figure 2, we decided to retrieve miRNAs that are both differently expressed in islets from IGT/T2D vs NGT donors and significantly correlated with HbA1c. This was performed to detect differentially regulated miRNAs that were more likely to have an impact on insulin secretion and, therefore, β-cell function. This analysis resulted in two distinct miRNA datasets. The first consisted of nine miRNAs that were upregulated in IGT/T2D islets and positively correlated with HbA1c levels (*Up-Pos*). The second contained 22 miRNAs that were downregulated in IGT/T2D islets and negatively correlated with HbA1c levels (*Down-Neg*). The nine miRNAs in the *Up-Pos* group were miR-1275, miR-629-5p, miR-513a-5p, miR-32-3p, miR-1236-3p, miR-1827, miR-130b-5p, miR-509-5p, and miR-130b-3p and among the miRNAs in the *Down-Neg* group were miR-200b-3p, miR-23b-3p, miR-19b-3p, miR-29b-3p, miR-200a-3p, miR-7-5p, and miR-126-3p (Figure 2).

### Gene target analysis of miRNAs with altered expression due to glycemic status

**Figure 3:**
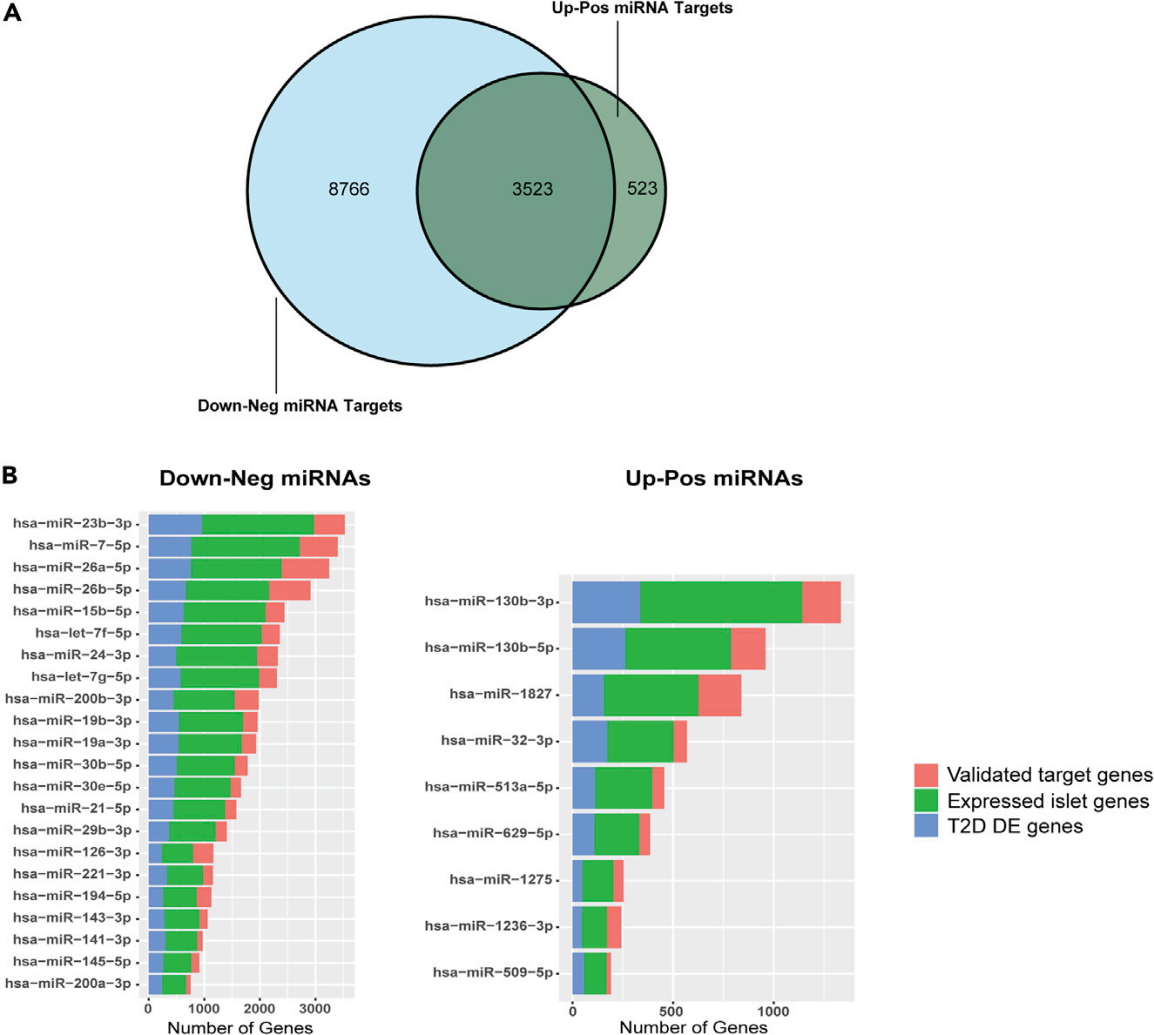
Gene target analysis of miRNAs with altered expression due to glycemic status (A) Venn diagram representing the overlap between validated target genes of the *Up-Pos* and *Down-Neg* miRNA sets. (B) Proportion of expressed islet genes and genes that overlap previously recorded T2D-related genes in regard to the total validated target genes of the *Down-Neg* (left) and *Up-Pos* (right) miRNA sets. Red bar = total validated genes; Green bar = Proportion of validated genes expressed in human islets; Blue bar = Proportion of validated genes overlapped with T2D differentially expressed genes.

We were curious to explore the pathways in which these two miRNA datasets (*Up-Pos* and *Down-Neg*) are involved and, consequently, altered in response to glycemic status. To this end, we first determined the gene targets of each miRNA by obtaining a list of experimentally validated gene targets for our miRNAs of interest. Since target validation of large miRNA sets has been facilitated by the introduction of high-throughput methods, validated gene targets of each miRNA were derived from Tarbase v.8 (Karagkouni et al., 2018) and miRTarBase v.7 (Chou et al., 2018) databases (Table S5). The nine *Up-Pos* miRNAs were associated with 4,046 unique genes forming 5,232 miRNA-mRNA interactions and the 22 *Down-Neg* miRNAs were associated with 12,289 unique genes forming 41,950 miRNA-mRNA interactions. The higher number of interactions can be explained by the fact that different miRNAs can target the same gene and a single gene can be targeted by multiple miRNAs (Eliasson and Esguerra, 2020). We could show that the same number of random miRNAs could not achieve similar target/interaction numbers (Figure S2A). The *Down-Neg* miRNAs shared 29% of their gene targets with the *Up-Pos* miRNAs (Figure 3A).

Furthermore, we clustered the miRNAs in the two datasets according to the similarity of their targets, in an attempt to determine miRNAs that work in a synergistic way. Using the Jaccard distance, which is based on the proportion of gene targets that are not shared between miRNAs, heatmaps of the identity plots for each dataset were created (Figure S2B). Similarities were observed in the *Down-Neg* dataset, in which we identified two functional clusters consisting of *let-7g-5p/let-7f-5p/miR-30e-5p/miR-30b-5p/miR-19a-3p/miR-19b-3p* and *miR-24-3p, miR-15b-5p, miR-7-5p,* and *miR-23b-3p*, respectively. In addition, we detected expected high similarity between miRNAs of the same family (miR-30e-5p/miR-30b-5p, miR-19a-3p/miR-19b-3p, miR-26a-5p/miR-26b-5p, and miR-200a-3p/miR-141-3p).

Considering that the validated gene target lists are collected from experiments in different cell types or tissues, we decided to retain only genes that are expressed in human islets for further investigation. To this end, we made use of the published human islet transcriptome data of 188 donors (Asplund et al., 2020). Expressed genes were considered those with at least three normalized counts in at least 80% of the samples, leaving a total of 11,689 out of the 60,517 identified genes (Table S6). We showed that a high percentage of the validated gene targets of each miRNA are expressed in the human islets (miRNA with lowest overlap 68% - highest overlap 89%) (Figure 3B).

Next, we compared identified miRNA gene targets in islets with genes that showed altered expression in T2D donor islets in published bulk- and single-cell RNA-seq data (Bugliani et al., 2013; Fadista et al., 2014; Gunton et al., 2005; Lawlor et al., 2017; Segerstolpe et al., 2016; Solimena et al., 2018; Taneera et al., 2012; Xin et al., 2016). We decided to include all 3,365 unique genes with altered expression in T2D from all studies (Table S7), as there was only a small overlap between the differentially expressed genes across the studies. This could be due to differences in RNA-profiling technologies (bulk sequencing, single-cell sequencing, and microarrays) and parameters (e.g. islet isolation procedure, sample number, statistical analysis, and variable control) of each study (Table S7). The overlap between all validated targets genes of the two datasets, as well as the proportion of these that are islet specific and differently expressed in T2D, is shown in Figure 3B. It is worth mentioning that miR-130b-3p in the *Up-Pos* dataset and miR-23b-3p in the *Down-Neg* dataset had the highest number of validated and expressed gene targets in islets. The targets of these two miRNAs also displayed the highest overlap with previously reported T2D differently expressed genes.

### miRNA gene target clustering and functional annotation reveals two islet-specific clusters

**Figure 4:**
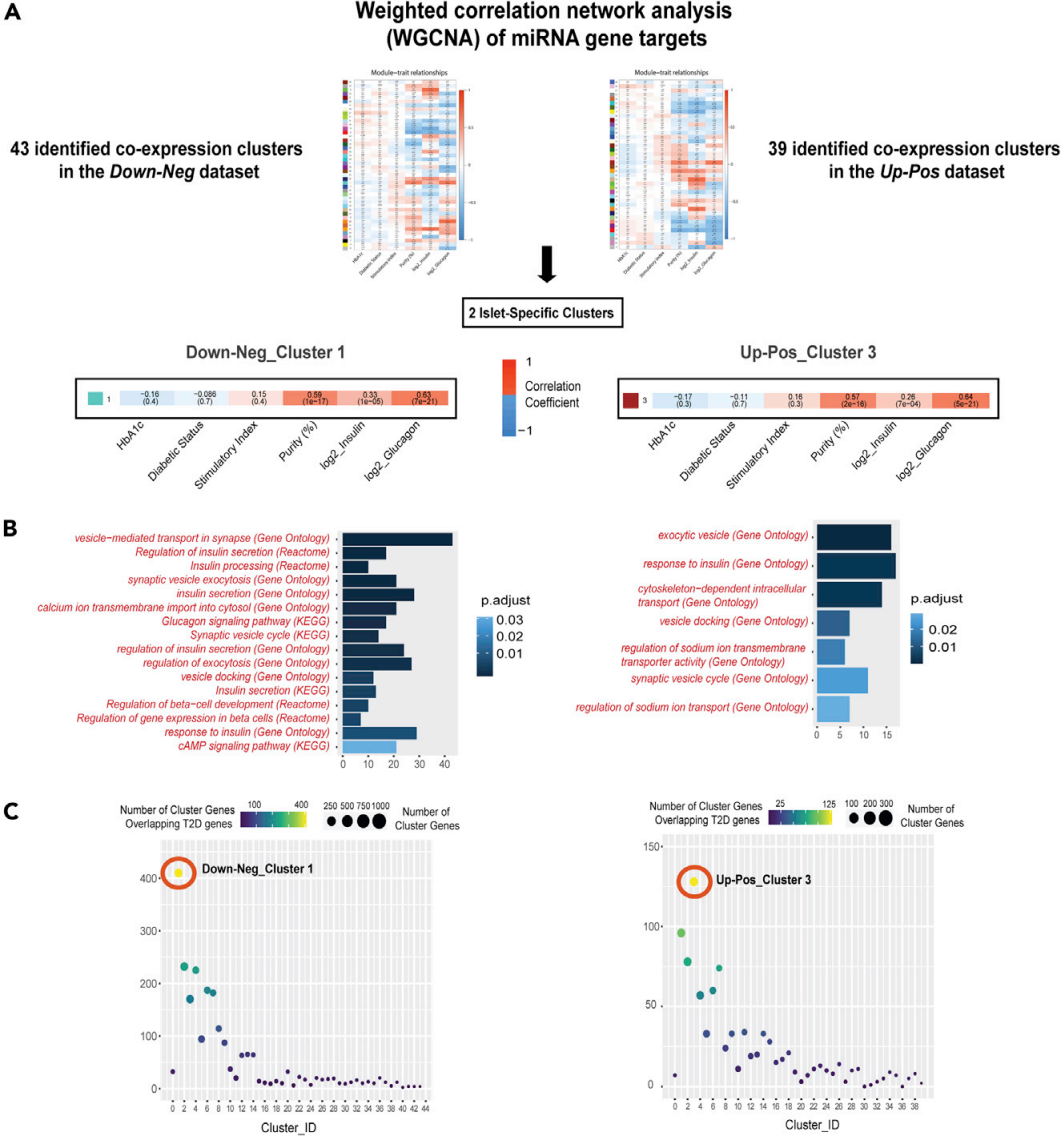
miRNA gene target clustering and functional annotation reveals two islet-specific clusters (A) WGCNA of miRNA gene targets and subsequent cluster correlation with phenotypic uncovered 2 clusters of interest, one for each of the *Up-Pos* and *Down-Neg* miRNA sets. Clusters (represented by both numbers and colors) were summarized to their eigengenes (first principal component) and were correlated with phenotypic traits. Each box illustrates the correlation coefficient, also represented by color scale, and the adjusted p values (in parentheses) of the correlation between the cluster eigengene and the corresponding trait value. (B) Enriched functional annotation terms and their database source are presented for Down-Neg_Cluster 1 (left) and Up-Pos_Cluster 3 (right). For each term, the number of genes associated with the term and the enrichment adjusted p value is indicated. (C) Bubble chart of all clusters of the *Down-Neg* (left) and the *Up-Pos* (right) miRNA gene target sets. Bubble size corresponds to the number of genes included in each cluster. The clusters are plotted against the number of overlaps with previously recorded differentially expressed genes in T2D, which is also represented by a color scale.

Next, we questioned whether miRNAs in the *Up-Pos* and *Down-Neg* datasets are implicated into processes related to β-cell function. We first clustered the genes into high-correlating groups (clusters) by performing a weighted gene correlation network analysis (WGCNA), as we wanted to identify gene co-expression networks regulated by miRNAs. This allowed us to divide the genes into 39 and 43 clusters of various sizes in the *Up-Pos* and *Down-Neg* datasets, respectively (Table S10). After summarizing the expression profile of the individual clusters using their eigengenes, which represent the first principal component, we were able to correlate each cluster with phenotypic traits such as HbA1c, diabetic status, stimulatory index, human islet purity, and the normalized expression (log2) of the insulin and glucagon genes. The selection of the expression levels of insulin and glucagon as traits enabled us to determine how α- or β-cell-enriched the clusters were. The *Up-Pos* dataset had 11 β-cell-enriched clusters, six α-cell-enriched clusters, and 17 clusters enriched for both α- and β-cells, while the *Down-Pos* dataset had 14 β-cell-enriched clusters, 14 α-cell-enriched clusters, and 13 enriched for both. As explained below, the two islet specific clusters which were further investigated were enriched for both α- and β-cell phenotypic traits (Figure 4A).

Enrichment analysis of functional annotation terms was then performed to associate the different clusters to biological pathways. The miRNA gene targets of each cluster were scanned for enriched Gene Ontology (GO) terms and terms belonging to the Reactome and KEGG pathway databases (Tables S8–S9). One cluster in the *Up-Pos* dataset (Up-Pos_Cluster 3) and one cluster in the *Down-Neg* dataset (Down-Neg_Cluster 1) included enriched terms/pathways related to α- and β-cells. In the *Up-Pos* dataset, we observed terms relative to exocytosis (“exocytotic vesicle”, “vesicle docking”) and “response to insulin”, while in the *Down-Neg* dataset we came across the term “insulin secretion” in all three databases, as well as pathways such as “insulin processing”, “regulation gene expression”, and “development in β-cells”, and terms related to responses to insulin and glucagon signaling pathways (Figure 4B).

Both Up-Pos_Cluster 3 and Down-Neg_Cluster 1 were significantly correlated with the purity of the islet samples (Cor.Coef = 0.57, adj. p value = 2 × 10^−16^; Cor.Coef = 0.59, adj. p value = 1 × 10^−17^). Moreover, both clusters were significantly correlated with the expression levels of insulin (Up-Pos_Cluster 3: Cor.Coef =0.26, adj. p value = 7 × 10^−4^; Down-Neg_Cluster 1: Cor.Coef =0.33, adj. p value = 1e - 05) and glucagon (Up-Pos_Cluster 3: Cor.Coef =0.64, adj. p value = 5 × 10^−21^; Down-Neg_Cluster 1: Cor.Coef =0.63, adj. p value = 7 × 10^−21^) in the islets. Other clusters also show significant correlation with islet purity, insulin, and glucagon levels, suggesting their involvement in other pathways inside the pancreatic islet.

Next, we compared all 82 clusters with the list of previously reported genes with altered expression in T2D (Table S7). Interestingly, Up-Pos_Cluster 3 and Down-Neg_Cluster 1 included a higher proportion of islet T2D genes (Up-Pos_Cluster 3: 42%, Down-Neg_Cluster 1: 40%) compared to other clusters of similar size in each dataset (Figure 4C, Table S10). Finally, we observed a broad specificity of differentially expressed miRNAs (Figure 2) regarding gene targeting in the different clusters, as almost all miRNAs in each dataset target genes belonging to medium-sized and large clusters (Figure S3).

### Genetic regulation of altered and abundant miRNAs

eQTLs can provide an important step to link the miRNAs with T2D-risk variations derived from previous GWAS studies (Buniello et al., 2019; Piñero et al., 2020; Ramos et al., 2014). We therefore sought to determine *cis*-eQTLs mapping to within a 1Mb window of the starting position of each miRNA for our miRNA sets. This analysis was performed in three different miRNA datasets: the *Up-Pos*, *Down-Neg*, and the 15 most abundant miRNAs (*top15*). No eQTL was found to be statistically significant after p value correction for multiple testing which could be explained by the low study power of the analysis (n=18). Given that multiple nominal signals at the same locus are suggestive of a link between the genetic variant and miRNA expression, we considered eQTLs showing a nominal p value<0.05 (Table S11).

**Figure 5:**
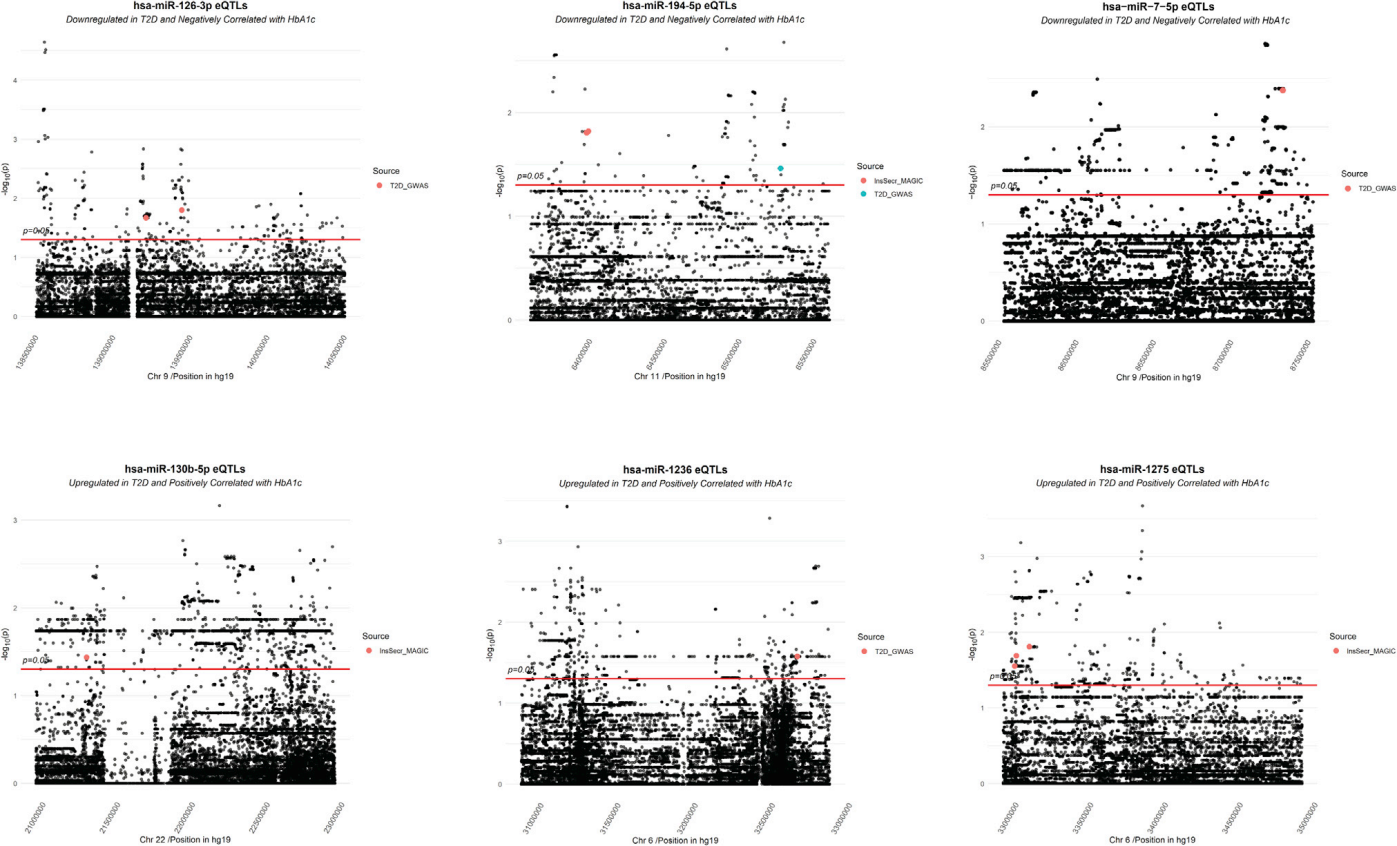
eQTL locus zoom plots of miRNAs with suggestive signals of association with insulin secretion/T2D-risk loci The points represent eQTLs within 1Mb of the start of the corresponding miRNA and are plotted against the significance of their correlation to the expression of the miRNA (-log10 p value). eQTLs above the red line have a nominal p value<0.05. Highlighted are the eQTLs that overlap T2D-risk variants or variants associated with insulin secretion traits. eQTLs in very close proximity may overlap, so it is recommended to also consider the complete list in Table S13.

Co-localization of the miRNA eQTLs with T2D-risk loci would provide additional hints of the implications of these miRNAs in the disease. After assembling GWAS data from multiple sources, we generated a list of SNPs that have been associated with T2D and related glycemic and insulin secretion traits (Methods and Table S12). The list was used to check for overlaps between these SNPs and the miRNA eQTLs in the three miRNA datasets. In total, three miRNAs (miR-7-5p, miR-126-3p, miR-1236) had nominal eQTLs overlapping with suggestive signals for T2D, two miRNAs (miR-130b-5p, miR-1275) had nominal eQTLs overlapping with suggestive signals for insulin secretion indices, and miR-194-5p had nominal eQTLs overlapping with both signals (Figure 5, Table S13).

## Discussion

In this study, we investigated miRNAs-mRNA networks in pancreatic islets and their potential role in the pathogenesis of T2D. We identified miRNAs with altered expression in hyperglycemia, as well as their target genes. We also explored clusters of islet miRNA-mRNA networks and identified specific clusters associated with insulin and glucagon expression as well as T2D. Moreover, we found suggestive eQTLs linking genetic variation to the differentially expressed miRNAs, which overlapped with T2D and insulin secretion GWAS signals.

The profiling of the miRNA expression levels in human islets revealed a limited number of highly expressed miRNAs, some of which have been previously described to be important for islet function. Among the 15 most expressed miRNAs, we identified miR-375, miR-29 family (miR-29a/b/c), members of miR-200 family (miR-200c/miR-141), and miR-7. miR-375 is important in the regulation of many functions in the β-cell including proliferation, insulin synthesis, processing, and secretion (Eliasson, 2017). Members of the miR-29 and miR-200 families are implicated in β-cell apoptosis and insulin secretion, and miR-7 is involved in insulin secretion and development (reviewed in (Eliasson and Esguerra, 2020; LaPierre and Stoffel, 2017)). The most abundant miRNA in our assay, miR-1246, has not previously been described in a β-cell context. However, it has been classified as a potential serum biomarker for diabetes (Vasu et al., 2019) and pancreatic cancer prognosis (Wei et al., 2020).

We identified 37 upregulated and 26 downregulated miRNAs in islets from IGT/T2D donors compared to NGT. Other studies have previously shown a similar pattern regarding the larger number of upregulated miRNAs in diabetes/obesity in human, mice, and rat models (Esguerra et al., 2011; Locke et al., 2014; Nesca et al., 2013; Zhao et al., 2009). Hemoglobin A1c (HbA1c) is used as a measure of the average blood glucose levels over the last 2–3 months. As a means to increase our confidence that the expression of the differentially expressed miRNAs is associated with the donor glycemic state, we selected those that also showed a direct correlation with HbA1c in the appropriate direction. Thus, we ended up with a set of nine miRNAs upregulated and positively correlated with HbA1c (*Up-Pos*) and a set of 22 miRNAs downregulated and negatively correlated with HbA1c (*Down-Neg*). Interestingly, only five out of the 31 differentially expressed miRNAs are among the most abundant ones (miR-141-3p, miR-21-5p, miR-29b-3p, miR-24-3p, and miR-7-5p; all downregulated), meaning that regulation and abundance do not necessarily coincide. Moreover, we found miRNAs in the *Up-Pos* and *Down-Neg* datasets that have been investigated in separate studies with a defective β-cell function perspective (Eliasson and Esguerra, 2020; LaPierre and Stoffel, 2017).

Instead of studying the effects of individual miRNAs on the β-cell functionality, we implemented a network approach, in which the whole list of differentially expressed miRNAs could point to potentially dysregulated pathways in donors with altered glycemic status. The first step was to match the miRNAs of both sets with validated gene targets from public sources. We focused on validated rather than predicted miRNA targets as, despite considerable advances in the field of miRNA target prediction, the predictive algorithms are still far from perfect due to the inconsistency in miRNAs pairing with their target genes (Helwak et al., 2013; Liu and Wang, 2019). The fact that the list of the miRNA with most targets (miR-23b-3p) includes 3,529 genes is a good example of how a few thousand miRNAs can regulate over 60% of protein-coding genes (Friedman et al., 2009).

After filtering out gene targets not expressed in human islets, we implemented WGCNA to identify clusters of highly correlated miRNA gene targets. Functional annotation revealed two interesting clusters, one in the *Up-Pos* and one in the *Down-Neg* dataset, that most likely constitute islet-specific networks as they were positively associated with islet purity, insulin, and glucagon expression levels. This was also supported by the enrichment of functional terms related to islet function, such as “insulin biosynthesis and secretion”, “β-cell development”, and “response to insulin”. Of interest was Down-Neg_Cluster 1, comprising 1,019 correlated genes in total. Among these, there were genes crucial for the β-cell development and function, such as *NEUROD1* (Gu et al., 2010)*, PAX6* (Gosmain et al., 2012)*, NKX2-2* (Doyle and Sussel, 2007)*, FOXO1* (Buteau and Accili, 2007), and *KCNB1* (Fu et al., 2017). Hence, gene interactions can produce a highly complex network that fine-tunes distinct and vital functions within the islet. However, only some of the genes in the Down-Neg_Cluster 1 was previously reported to be differentially expressed in T2D (410/1,019 genes) (Bugliani et al., 2013; Fadista et al., 2014; Gunton et al., 2005; Lawlor et al., 2017; Segerstolpe et al., 2016; Solimena et al., 2018; Taneera et al., 2012; Xin et al., 2016) This can be explained by the disease module hypothesis, wherein a functional gene association will not fundamentally correspond to a specific disease phenotype (Barabási et al., 2011), meaning that dysregulation of only some of the genes in the module (cluster) can potentially be implicated in the pathophysiology of T2D.

GWAS facilitate the identification of disease-risk loci through associating genetic variants to diseases such as T2D. These loci are named after the nearest gene, which may not necessarily be the causal gene. In order to identify the causal gene, these genetic variants are investigated for their association with gene or miRNA expression, so called expression quantitative trait loci (eQTLs). Mapping miRNA expression to proximal loci did not result in any significant associations, potentially due to the small power of our study (n=18). However, even larger-scale studies did not manage to identify statistically significant eQTLs. In a previous study with 176 lymphoblastoid cell lines, no *cis*-miR-eQTLs were found (Gamazon et al., 2012), while another study in primary fibroblasts of 180 new-borns revealed only 12 *cis*-miR-eQTLs with FDR<0.5 (Borel et al., 2011). The complications of correlating SNPs to miRNA is confirmed by a large-scale study, which reports that single *cis*-miRNA eQTLs can explain only a small portion of the expression variability of their associated miRNAs (1.3%) compared to the *cis*-mRNA eQTLs, which explain 33%–53% of variance in the expression levels of their associated mRNAs (Huan et al., 2015). This implies that there might be more suitable statistical models, other than linear correlations, that could identify *cis*-miRNA-eQTLs with more precision.

However, even multiple suggestive signals linking genetic variation to miRNA expression, which in turn is correlated to a relevant phenotype, can be indicative of their involvement in that particular phenotype. The overlap between 13 miRNA eQTLs with T2D loci and insulin secretion indices suggests the implication of differentially expressed miRNA regulating genes of *Up-Pos* and *Down-Neg* datasets in T2D. Specifically, eQTL of miR-194 overlaps with suggestive signals for both T2D and insulin secretion phenotypes. This miRNA has previously been shown to be a biomarker for diabetes incidence (Jaeger et al., 2018), as well as a regulator of glucose metabolism in skeletal muscle (Latouche et al., 2016). Another interesting miRNA, miR-126, whose eQTLs showed suggestive signals for T2D, was found to be a potent biomarker for early prognosis of diabetes (Liu et al., 2014) and acts as a protective agent against diabetic vascular complications (Suresh Babu et al., 2016). It is worth mentioning that the eQTLs of miR-1236, despite demonstrating a suggestive signal for T2D, also show overlaps with 15 SNPs which were linked to “insulin-dependent diabetes” (Piñero et al., 2020), an interesting finding considering the role of miR-1236 as a biomarker for latent autoimmune diabetes in adults (LADA) (Yu et al., 2019). Out of the two miRNAs whose eQTL profile suggests signals for insulin secretion traits, miR-130b has been shown to affect intracellular ATP levels in the pancreatic β-cell, indicating a possible effect on insulin secretion (Ofori et al., 2017), while miR-1275 has not been associated with T2D or insulin secretion before. Notably, none of the 13 eQTLs with suggestive T2D-risk/insulin secretion signals has been functionally linked to T2D so far, making them suitable candidates for future T2D studies. Moreover, additional eQTLs of the six miRNAs with strong nominal signals that have not been associated with any existing traits before could be considered potential targets of interest in the pursuit of a more complete comprehension of T2D etiology.

In conclusion, our work demonstrates the complexity of the miRNA-mRNA network regulation in pancreatic islets. Therefore, efforts toward miRNA-based therapeutic strategies should focus on the comprehension of the role of individual miRNAs as network components rather than individual gene modulators.

### Limitations of the study

The differentially regulated miRNAs identified in this study do not overlap with those described in other studies that compare healthy and T2D human islets (Kameswaran et al., 2014; Locke et al., 2014). The variability of individual donors in terms of their underlying pathophysiological condition, the differences in islet isolation and culturing techniques, the small number of samples under comparison and the distinct profiling methods could all provide plausible explanations for this discrepancy. Another caveat is the small number of samples included in the study, a common problem due to the limited supply of human islets. This is why the principal aim of this study is to explore the complexity of the miRNA-mRNA networks and how they can potentially contribute to T2D onset, rather than defining casual miRNA-mRNA-phenotype relationships. Moreover, miRNA-regulated network analysis in islets was based on the collection of experimentally validated miRNA gene target data from multiple studies. However, it should be noted that despite revealing genuine miRNA-mRNA interactions, high-throughput miRNA-target validation techniques are not necessarily accompanied by functional target regulation (Liu and Wang, 2019) and results should be interpreted with caution.

## STAR★Methods

### Key resources table

**Table:** 

REAGENT or RESOURCE	SOURCE	IDENTIFIER
**Biological samples**		

Human pancreatic islets from healthy donors and donors with T2D	Nordic Network for Clinical Islet Transplantation, Human Tissue Laboratory, EXODIAB/LUDC	N/A

**Chemicals, peptides, and recombinant proteins**		

miRCURY Hy3 fluorescent dye	Exiqon	208032-A

**Critical commercial assays**		

miRNeasy	Qiagen	217004
miRCURY LNA microRNA array v.11.0	Exiqon	208202-A
Infinium OmniExpress-24 v1.3	Illumina	20024632

**Deposited data**		

Raw and processed miRNA microarray sequencing and differential miRNA expression data	This paper	ArrayExpress (E-MTAB-11125)

**Software and algorithms**		

Adobe Illustrator	Adobe	https://www.adobe.com/products/illustrator.html, RRID: SCR_010279
Genepix Pro 4.1	Molecular Devices	RRID: SCR_ 010969
CARMAweb 1.4	Rainer et al. (2006)	https://carmaweb.genome.tugraz.at/carma/
miRBaseConverter 1.12.0	Bioconductor	http://bioconductor.org/packages/release/bioc/html/miRBaseConverter.html
multiMiR 2.3.0	Bioconductor	http://bioconductor.org/packages/release/bioc/html/multiMiR.html
WGCNA 1.70-3	Comprehensive R Archive Network (CRAN)	https://horvath.genetics.ucla.edu/html/CoexpressionNetwork/Rpackages/WGCNA/, RRID:SCR_003302
FastQTL 2.0	FunPopGen lab, University of Geneva	http://fastqtl.sourceforge.net/
LiftOver	University of California, Santa Cruz	https://genome.ucsc.edu/cgi-bin/hgLiftOver
Michigan Imputation Server	Das et al. (2016)	https://imputationserver.sph.umich.edu
Eagle v2.4	Loh et al. (2016)	https://alkesgroup.broadinstitute.org/Eagle/

### Resource availability

#### Lead contact

Further information and requests for resources and reagents should be directed to and will be fulfilled by the lead contact Lena Eliasson (lena.eliasson@med.lu.se).

#### Materials availability

This study did not generate new unique reagents.

### Experimental model and subject details

Human pancreatic islets from non-diabetic and T2D donors with varying levels of HbA1c were obtained from the Nordic Network for Clinical Islet Transplantation and the Human Tissue Laboratory, EXODIAB/LUDC. Donor characteristics (sex, age, BMI, HbA1c levels, glycemic status) are presented in Table S1. Donors or their relatives had given their written consent to donate organs for biomedical research upon admission to the intensive care unit. The work was approved by ethics committees at Uppsala and Lund Universities. The islets were processed as previously described (Andersson et al., 2012) and handpicked under stereomicroscope before use. The 18 donors were divided into three experimental groups according to their HbA1c levels. The HbA1c of the 18 donors ranges from 4.6% to 7%, 7 had normal glucose tolerance (NGT) (HbA1c<6%), 6 had impaired glucose tolerance (IGT) (HBA1c range: 6.0 - 6.4%), while 5 of them were also diagnosed with T2D (HbA1c range: 6.2 - 7.0%).

### Method details

#### Locked nucleic acid (LNA)-based microarray of human microRNAs

Total RNA from handpicked human islets was extracted using miRNeasy kit (Qiagen). RNA quantity and quality were evaluated using spectrophotometry by Nanodrop and electropherogram profiles by Experion (BioRad), respectively. 500 ng of total RNA was directly labelled with miRCURY Hy3 fluorescent dye in the power labelling kit (#208032-A, Exiqon). The labelled RNA samples were subsequently hybridized to miRCURY LNA microRNA array v.11.0 (#208202-A, Exiqon) in a Maui hybridisation chamber according to manufacturer’s recommendations. LNA-modified capture probes exhibit enhanced hybridisation properties, with results comparable to next-generation sequencing approaches to expression profiling (Willenbrock et al., 2009). Images were acquired at 10 um resolution using Agilent array scanner (G2505C), and spot intensities were quantified in Genepix Pro 4.1. Array signals were normalized using the global lowess regression algorithm as implemented in CARMAweb 1.4 (Rainer et al., 2006). miRNA name annotation across different miRBase versions was performed with the R package miRBaseConverter (v. 1.12.0) (Xu et al., 2018).

#### miRNA-gene interactions

Gene targets of miRNA sets were identified using multiMiR (v. 2.3.0) with default settings (Ru et al., 2014), an R package that provides access to 14 external miRNA-gene interaction databases. Validated miRNA targets were considered the subset of all targets that originate from the Tarbase v.8 (Karagkouni et al., 2018) and miRTarBase v.7 (Chou et al., 2018) databases (Table S5).

#### Weighted gene correlation network analysis (WGCNA) analysis and cluster functional annotation

To construct a co-expression network of the miRNA target genes using their gene expression values as co-expression measure, the R package WGCNA (v. 1.70-3) was used (Langfelder and Horvath, 2008). The gene expression values of the targets derived from publicly available human islet transcriptomic data of 188 diseased donors (Asplund et al., 2020) and were log2 transformed for the analysis. Genes were considered expressed in human islets and were included in the analysis only if they had more than 3 normalized counts in at least 80% of the donors. After clustering the donors based on their Euclidean distance, one donor was considered an outlier and was excluded from subsequent analysis. A signed network was generated using the blockwiseModules function of the WGCNA package with a soft-thresholding power of 12 and with parameters reassignThreshold = 0, minModuleSize = 10, mergeCutHeight = 0.25, while the default settings were selected for the rest of the parameters. Functional annotation of the genes belonging to the corresponding clusters was then performed with hypergeometric testing using the R package clusterProfiler (v.3.16) for Gene Ontology and KEGG pathway terms (Yu et al., 2012), as well as the ReactomePA for Reactome pathway terms (Yu and He, 2016). Significantly enriched functional terms included those with an adjusted for multiple testing p-value<0.05 (Benjamini–Hochberg method). Correlation coefficients between the summary profile of the cluster (eigengene) expression (log-transformed) and phenotypes (traits) were computed using Pearson’s correlations for normally distributed traits (log2 insulin and log2 glucagon expression, HbA1c) and Spearman correlation for the non-normally distributed ones (diabetic status, islet purity).

#### *cis*-eQTL mapping

##### Imputation

Genotyping was performed using the Infinium OmniExpress-24 v1.3 kit (Illumina, #20024632). GWAS data was quality controlled as described previously (Asplund et al., 2020). Briefly, SNP exclusion criteria included missingness threshold of >0.05%, MAF<1% and Hardy-Weinberg equilibrium p value<0.05. Imputation was performed on the Michigan Imputation Server using HRC r1.1 (GRCh37/hg19) as a reference panel along with Eagle v2.4 phasing and “mixed” as population type (Das et al., 2016; Loh et al., 2016).

##### eQTL mapping

To identify *cis*-eQTLs of the miRNAs of interest FastQTL (v.2.0) was used (Ongen et al., 2016). After normalizing miRNA expression data with log2 transformation, the associations between genotype alleles and miRNA expression were calculated using a linear model with age, gender, BMI, HbA1c, and number of days islets were in culture as covariates. The search was restricted to variants within 1Mb of the starting position of each miRNA and p value adjustment for multiple testing was performed with the Benjamini & Hochberg correction.

##### Comprehensive catalogue of T2D-risk and related glycemic trait SNPs

Well-characterized T2D-risk variants discovered in multiple GWAS studies were acquired from 3 different publicly available databases: GWAS catalogue v1.0 (Buniello et al., 2019), DisGeNET v7.0 (Piñero et al., 2020) and PheGenI (as of June 2021) (Ramos et al., 2014). A comprehensive catalogue of 6,741 T2D-pathogenicity related variants was created by selecting variants according to keywords relating to “type-2 diabetes”, “glucose homeostasis” and “insulin levels” (Table S12). The coordinates of the list were converted to coordinates of the hg19 genomic build with the UCSC LiftOver tool (https://genome.ucsc.edu/cgi-bin/hgLiftOver). Moreover, variants associated with insulin secretion traits during OGTT (Prokopenko et al., 2014) have been processed and downloaded from www.magicinvestigators.org. Specifically, variants associated with insulin response (CIR), disposition index (DI) and HOMA-B with a nominal p value<10^-3^ were compiled in a list and included in the analysis (Table S12).

### Quantification and statistical analysis

#### GSIS in different donor groups

GSIS fold change values across the three donor groups (high/low glucose concentration) were compared with the Kruskal–Wallis test. The test did not reveal significant differences between the groups.

#### Correlation between miRNA expression and hormone gene expression

The expression of miRNA and genes was correlated using Pearson’s correlation in the log2-transformed values of both miRNA and mRNA data. The transformation ensures that data follow the normal distribution as validated by QQ-plots and the Shapiro-Wilk test. Significant correlations were considered those with a nominal p value<0.05.

#### Relationship between miRNA expression and HbA1c levels

In order to investigate the relationship between miRNA expression and HbA1c levels, we used linear regression models with HbA1c, age, BMI, gender, diabetes status (non-diabetic, impaired glucose tolerant, diabetic) and the duration of culture of the islets as covariates. In the models, the values of the 470 expressed miRNAs were log2 transformed and the normality of HbA1c (%) values was validated with a QQ-plot and the Shapiro-Wilk test. False discovery rate (FDR) analysis was performed for the p values regarding a possible influence of HbA1c on miRNA expression in order to handle multiple hypothesis testing, with a FDR q-value of 0.1.

## Data Availability

• Raw and processed miRNA microarray sequencing and differential miRNA expression data have been deposited at ArrayExpress and are publicly available as of the date of publication. Accession numbers are listed in the key resources table. • This paper does not report original code. • Any additional information required to reanalyze the data reported in this paper is available from the lead contact upon request.

## Supplemental information

Document S1. Figures S1–S3

Table S1. Donor Characteristics, related to Figures 1 and 2

Table S2. Global miRNA LNA-array based profiling of human islets, related to Figure 1

Table S3. miRNA correlations with mRNA of pancreatic hormones, related to Figure 1

Table S4. Differential miRNA expression due to glycemic status & correlation with HbA1c, related to Figure 2

Table S5. Differentially expressed miRNA validated gene targets, related to Figure 3

Table S6. Differentially expressed miRNA gene targets expressed in human islets, related to Figure 3B

Table S7. Previously reported T2D DE genes & studies summary, related to Figure 3B

Table S8. Up_Pos WGCNA Clusters Functional Annotation, related to Figure 4A

Table S9. Down_Neg WGCNA Clusters Functional Annotation, related to Figure 4A

Table S10. Cluster info & Overlaps with T2D-related genes and miRNA targeting, related to Figure 4C

Table S11. Significant eQTLs per miRNA set, related to Figure 5

Table S12. Insulin secretion and T2D-risk variant catalogs, related to Figure 5

Table S13. eQTL overlap with insulin secretion/T2D-risk variants, related to Figure 5
