# Supplementary material for: Human pancreatic islet miRNA-mRNA networks of altered miRNAs due to glycemic status
Source: iScience. 2022 Feb 26;25(4):103995. doi: 10.1016/j.isci.2022.103995 (PMC8927907; doi:10.1016/j.isci.2022.103995)
Supplement: Document S1. Figures S1–S3 [file mmc1.pdf]

iScience, Volume *25*

Supplemental information

Human pancreatic islet miRNA-mRNA networks

of altered miRNAs due to glycemic status

Alexandros Karagiannopoulos, Jonathan L.S. Esguerra, Morten G. Pedersen, Anna Wendt, Rashmi B. Prasad, and Lena Eliasson

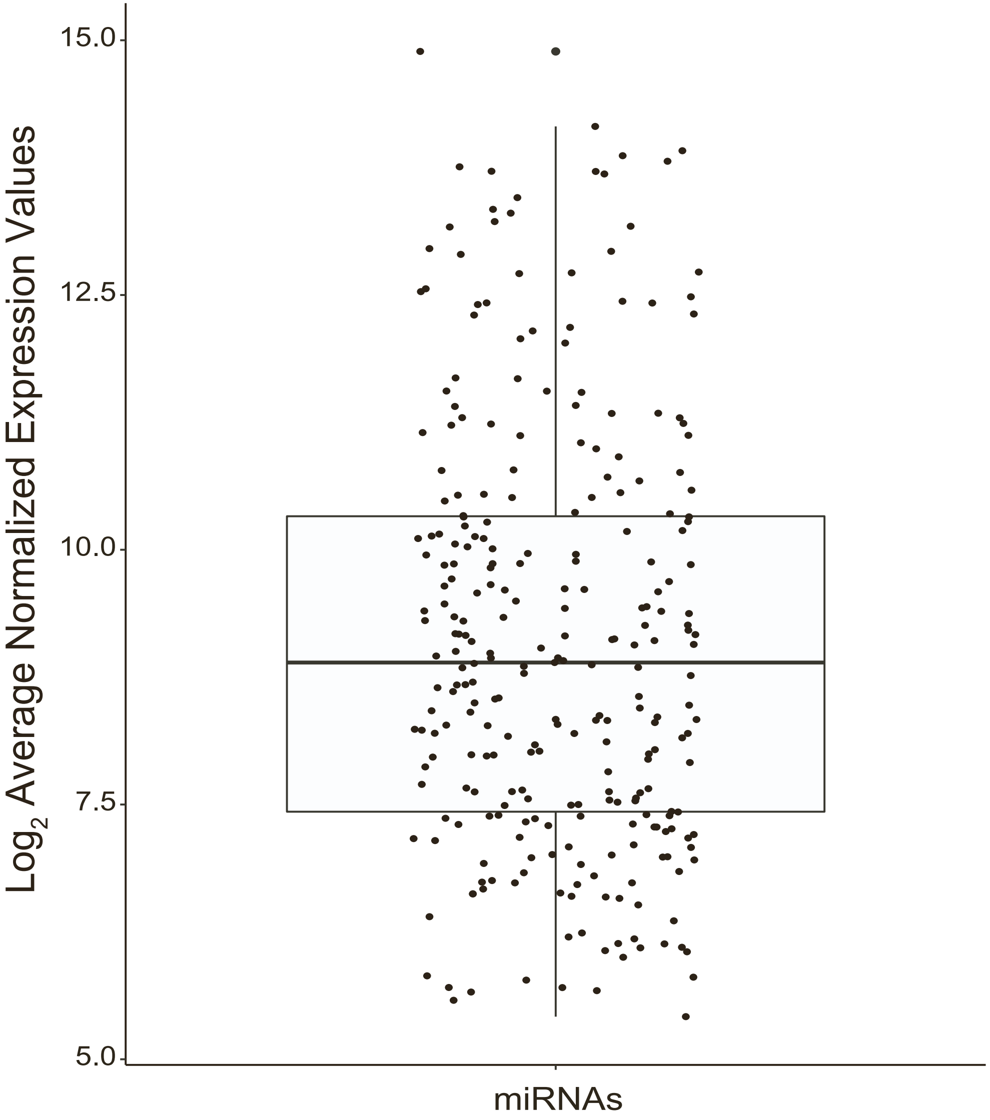

<u>**Figure S1.**</u> **Global miRNA profiling of human pancreatic islets.** Distribution of miRNA expression levels across all donors. Related to Figure 1.

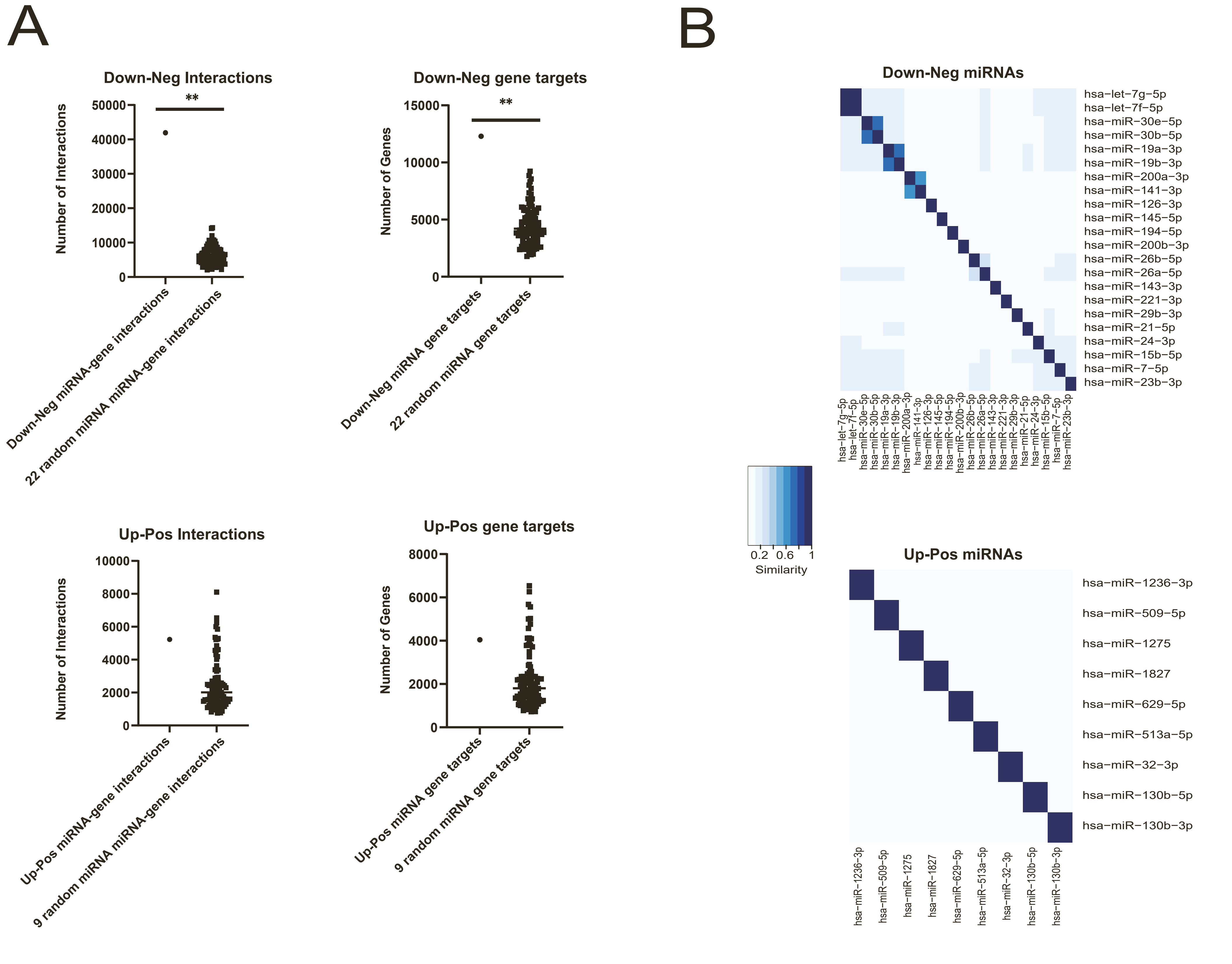

__Figure S2.__ **Further gene target analysis of miRNAs with altered expression due to glycaemic status.** (A) Comparison of the number of target genes of the 9 Up-Pos and the 22 Down-Neg miRNA data sets with a set of 9 and 22 random miRNAs respectively, after repeating the procedure 100 times. (B) Heatmap of the identity plot of the Up-Pos and Down-Neg miRNA sets, illustrating the similarity of the miRNAs based on their mutual gene targets represented by their Jaccard distance. Related to Figure 3.

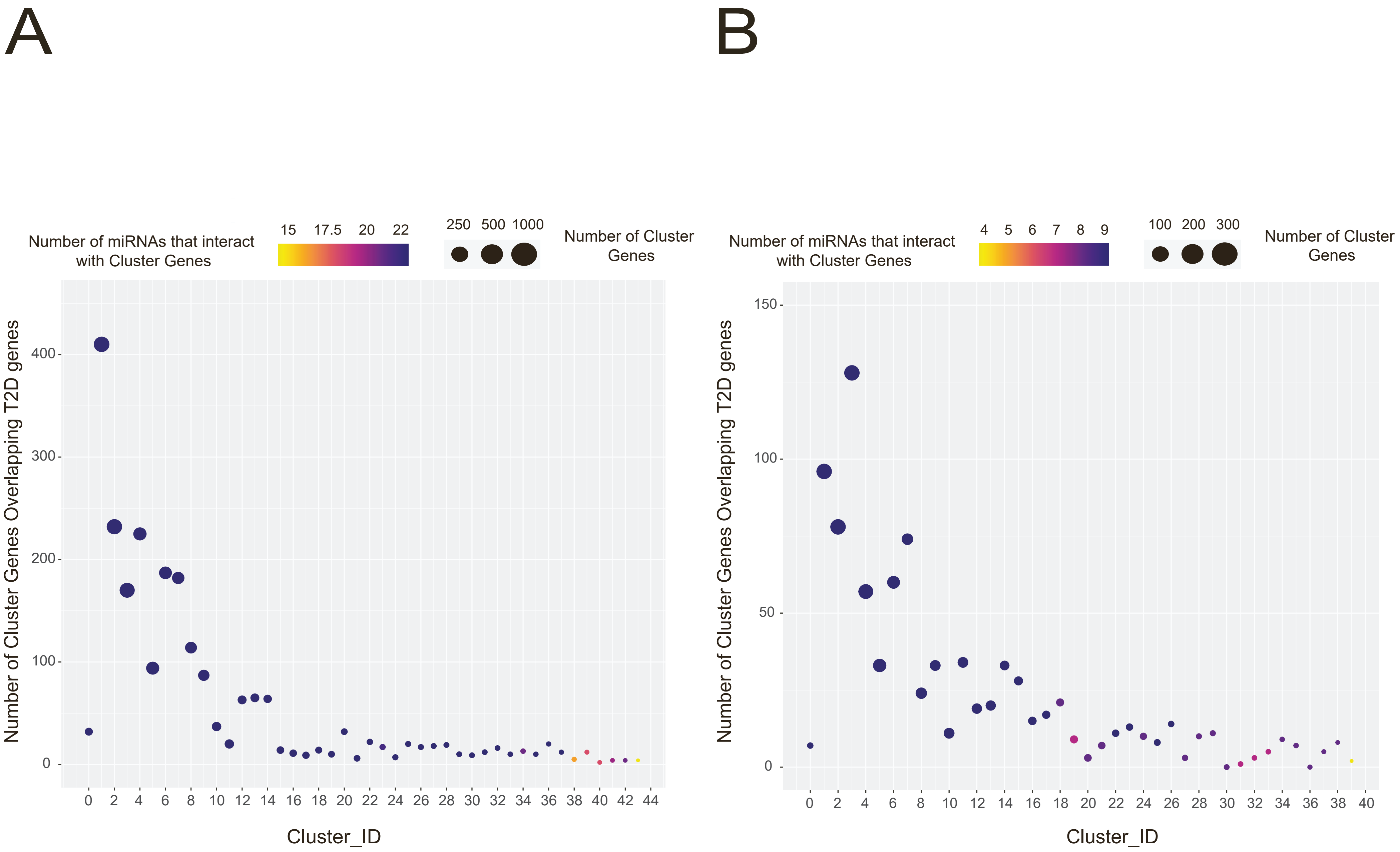

Figure S3. Broad specificity of differentially expressed miRNAs regarding gene targeting in the different clusters. Bubble chart of all clusters of the Down-Neg (A) and the Up-Pos (B) miRNA gene target sets. Bubble size corresponds to the number of genes included in each cluster. The clusters are plotted against the number of overlaps with previously recorded differentially expressed genes in T2D. The color scale represents the total number of miRNAs targeting at least one gene in the corresponding clusters. Related to Figure 4.
